# *Baylisascaris procyonis* Roundworm Seroprevalence among Wildlife Rehabilitators, United States and Canada, 2012–2015

**DOI:** 10.3201/eid2212.160467

**Published:** 2016-12

**Authors:** Sarah G.H. Sapp, Lisa N. Rascoe, Patricia P. Wilkins, Sukwan Handali, Elizabeth B. Gray, Mark Eberhard, Dana M. Woodhall, Susan P. Montgomery, Karen L. Bailey, Emily W. Lankau, Michael J. Yabsley

**Affiliations:** University of Georgia, Athens, Georgia, USA (S.G.H. Sapp, K.L. Bailey, E.W. Lankau, M.J. Yabsley);; Centers for Disease Control and Prevention, Atlanta, Georgia, USA (L.N. Rascoe, P.P. Wilkins, S. Handali, E.B. Gray, M. Eberhard, D.M. Woodhall, S.P. Montgomery);; Kentucky Wildlife Center, Lexington, Kentucky, USA (K.L. Bailey)

**Keywords:** Baylisascaris procyonis, baylisascariasis, Ascaridoidea, ascarid roundworm, seroprevalence, wildlife, wildlife rehabilitators, larva migrans, zoonoses, raccoons, occupational illnesses, United States, Canada, parasites, roundworms

## Abstract

*Baylisascaris procyonis* roundworms can cause potentially fatal neural larva migrans in many species, including humans. However, the clinical spectrum of baylisascariasis is not completely understood. We tested 347 asymptomatic adult wildlife rehabilitators for *B. procyonis* antibodies; 24 were positive, suggesting that subclinical baylisascariasis is occurring among this population.

*Baylisascaris procyonis*, a roundworm of raccoons (*Procyon lotor*) and rarely dogs, can cause fatal neural larva migrans or ocular larval migrans in numerous bird and mammal species, including humans (*1*). At least 54 human cases have been reported; however, cases may not have been recognized or reported, especially ocular cases, for which parasite identification is rare (*1*–*3*). Most diagnosed cases have been in children and were severe or fatal. Treatment is difficult after onset of neurologic symptoms, and neural larva migrans survivors may have permanent neurologic sequelae (*1*).

The clinical spectrum of baylisascariasis is not fully understood. Limited evidence suggests that subclinical disease may occur (*1*,*2*,*4*,*5*). *Baylisascaris* larvae were an incidental finding in the brain of an Alzheimer disease patient (*4*), and *B. procyonis* antibodies were reported in the parents of a child with baylisascariasis and in 4 of 13 adults in Germany with raccoon contact; assay specificity was not reported (*2*,*5*). The occurrence of subclinical infections with related ascarids (e.g., *Toxocara* species) is well established; up to 14% of persons in the United States are seropositive, although it is unknown how many have clinical manifestations (*6*).

Wildlife rehabilitators may represent a population at risk for subclinical baylisascariasis due to frequent contact with raccoons and their feces, which may contain infectious larvated *B. procyonis* eggs. We assessed the occurrence of antibodies to *B. procyonis* in a sample of wildlife rehabilitators from the United States and Canada and administered a questionnaire on rehabilitation experience and procedures.

## The Study

During 2012–2015, we collected serum samples from and administered questionnaires to wildlife rehabilitators (details in Technical Appendix). We tested serum samples for *B. procyonis* IgG using a recombinant *B. procyonis* repeat antigen 1 protein Western blot as described (*7*).

Of 347 enrolled persons (Table 1), 315 (91%) reported current involvement in rehabilitation activities. Participants had an average of 10.5 (median 7.0) years of animal rehabilitation experience. Most respondents (92%) reported having contact with raccoons at some point; 64% reported actively rehabilitating raccoons in the past year (Table 2).

**Table 1:** Demographic characteristics of participants in a study of *Baylisascaris procyonis* roundworm seroprevalence among wildlife rehabilitators, United States and Canada, 2012–2015

Variable	No. (%) respondents, N = 347	No. (%) seropositive
Sex		
Female	299 (86.2)	21 (7.0)
Male	48 (13.8)	3 (6.3)
Race		
Asian	6 (1.7)	0
American Indian or Alaska Native	1 (0.3)	0
Black or African American	1 (0.3)	0
White	327 (94.2)	23 (7.0)
Other	2 (0.6)	0
Multiracial	10 (2.9)	1 (10.0)
Ethnicity		
Hispanic	5 (1.4)	0
Not Hispanic	315 (90.8)	19 (6.0)
Declined to state	27 (7.8)	5 (18.5)
Geographic region of rehabilitation activities*		
Northeastern	106 (30.5)	4 (3.8)
Midwestern	74 (21.3)	8 (10.8)
Central	23 (6.6)	0
Southern	110 (31.7)	5 (4.5)
Western	34 (9.8)	7 (20.6)

*Geographic regions are defined as follows: Northeastern: Delaware, Maryland, Massachusetts, Maine, New Jersey, New York, Pennsylvania, and Virginia, USA, and Quebec Province, Canada; Midwestern: Illinois, Indiana, Kentucky, Michigan, Minnesota, Missouri, Ohio, and Wisconsin , USA, and Manitoba and Ontario Provinces, Canada; Central: Arizona, Colorado, Kansas, Oklahoma, and Texas, and Alberta, Province, Canada; Southern: Alabama, Florida, Georgia, Louisiana, Mississippi, North Carolina, South Carolina, and Tennessee, USA; and Western: California, Oregon, and Washington, USA, and British Columbia Province, Canada.

**Table 2:** Rehabilitation work characteristics and experience of wildlife rehabilitators enrolled in a study of *Baylisascaris procyonis* roundworm seroprevalence among wildlife rehabilitators, United States and Canada, 2012–2015

Variable	No. (%) respondents	No. (%) seropositive
Involvement in wildlife rehabilitation, N = 347		
Currently involved	314 (90.5)	22 (7.0)
Formerly involved	19 (5.5)	0 (0)
Other raccoon contact	14 (4.0)	2 (14.3)
Rehabilitation experience, y, N = 322		
<2.0	48 (14.9)	2 (4.2)
2.0–4.9	96 (29.8)	7 (7.3)
5.0–9.9	67 (20.8)	1 (1.5)
10.0–20.0	64 (19.9)	8 (12.5)
>20.0	47 (14.6)	3 (6.4)
Raccoon rehabilitation, N = 347		
Rehabilitated raccoons in past year	222 (64.0)	16 (7.2)
Rehabilitated raccoons (prior to past year)	41 (11.8)	2 (4.9)
Never rehabilitated raccoons	84 (24.2)	6 (7.1)
General raccoon contact, N = 329		
Had contact in past year	266 (80.9)	19 (7.1)
Had contact ever	36 (10.9)	3 (8.3)
Never had contact	27 (8.2)	2 (7.4)
*B.* *procyonis* prevalence among raccoons in state or province of residence, N = 347*		
Very high (>50%)	79 (22.8)	14 (21.5)
High (25%–49%)	127 (36.6)	5 (4.6)
Medium (10%–24%)	92 (26.5)	4 (4.3)
Low (<10%), sporadic, or unknown	49 (14.1)	1 (2.1)

*Prevalence levels in the various US states and Canadian Provinces are shown in the Figure.

Twenty-four (7%; 95% CI 4.7%–10.1%) participants tested positive for *B. procyonis* antibodies; adjusted prevalence, considering assay performance characteristics, was 5.7% (95% CI 2.2%–9.2%) (Figure) (*12*). Of those 24 participants, 22 (92%) were actively rehabilitating wildlife; the other 2 reported occasional wildlife contact, including contact with raccoons, through veterinary clinic activities. All but 2 seropositive persons reported raccoon contact, and 2 practiced rehabilitation in the same household. Nineteen (79%) of the 24 seropositive persons resided in a US state or Canadian province classified as having very high or high *B. procyonis* prevalence among raccoons (Table 2).

**Figure:**
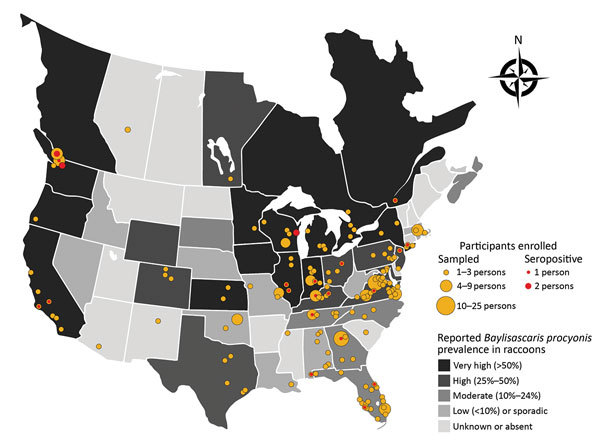
Locations for participant sampling in a study of *Baylisascaris procyonis* roundworm seroprevalence among wildlife rehabilitators, United States and Canada, 2012–2015. Yellow dots indicate counties (USA) or township/municipality (Canada) in which enrolled persons reported practicing wildlife rehabilitation. Red dots indicate locations of seropositive persons. Shading of states/provinces indicates general state/province level prevalence of *B. procyonis* in raccoons based on published reports (*1*,*8*–*11*).

## Conclusions

We detected antibodies to *B. procyonis* roundworms in 7% of wildlife rehabilitators we tested, suggesting that exposure to this zoonotic parasite may occur without clinical disease. Participants reported various degrees of raccoon contact. Although the transmission source could not be determined (i.e., from rehabilitation of raccoons or from exposure to eggs during other activities), use of gloves and handwashing was generally inconsistent among the seropositive persons in this study (S.G.H. Sapp, data not shown). *B. procyonis* is transmitted by ingestion of larvated eggs; thus, proper use of personal protective equipment (PPE), adherence to cleaning and disinfection protocols, and proper hand hygiene should minimize the risk associated with exposure to feces.

Transmission risk can also occur when handling animals whose fur has been contaminated by infective raccoon eggs, as shown for *Toxocara canis* parasites and dog fur (*13*). More investigations are needed regarding the occurrence of *B. procyonis* eggs on raccoon fur and transmission implications. Lapses in PPE use and hand hygiene may indicate a lack of caution or risk awareness for other pathogens.

Wildlife rehabilitators in areas with a very high prevalence of *B. procyonis* infection among raccoons may be at elevated risk for subclinical infections. Only 1 *B. procyonis*–seropositive wildlife rehabilitator resided in a state with low or sporadic prevalence (Alabama); however, that person lived in an area adjacent to a Florida county where the prevalence of *B. procyonis* infection in raccoons was 9% (M.J. Yabsley, unpub. data) (Figure). Data on *B. procyonis* prevalence in raccoons are outdated or missing for many US states and Canadian provinces. Furthermore, raccoon infections with *B. procyonis* are now being reported in areas where the parasite has historically been absent (e.g., the southeastern United States); thus, awareness of this parasite may be limited in those areas (*8*). More surveillance is needed on the distribution and prevalence of *B. procyonis* infection among raccoons to assess the association with exposure risks among humans.

Rehabilitation facilities housing raccoons can easily be contaminated with *B. procyonis* because high numbers of environmentally hardy eggs are passed by infected raccoons (*1*). Our finding of 2 seropositive raccoon rehabilitators operating out of the same household highlights the importance of infection-control practices. Facility contamination can be prevented by treating raccoons for parasites at intake and at regular intervals thereafter and by sterilizing enclosures using heat-based methods (*14*). Several anthelmintic drugs can kill adult *B. procyonis*, but raccoons with high worm burdens may require retreatment (*15*). Raccoon enclosures and housing should be constructed with materials that are easy to clean and disinfect using heat-based methods.

We tested persons with wildlife (mostly raccoon) contact, so our results describe an exposure risk that likely does not apply to the general public. However, persons in other occupations or activities (e.g., zoo keepers, wildlife biologists) may have similar exposure risks. Domestic dogs, other wildlife species (e.g., skunks, bears), and some exotic pets (e.g., kinkajous) are hosts for *Baylisascaris* spp. parasites and may present exposure risks (*1*). Although the assay we used has a sensitivity of 88% and specificity of 98%, it is time-consuming and not ideal for large-scale epidemiologic studies (*7*). Development of a high-quality ELISA would facilitate larger epidemiologic studies on the risk for baylisascariasis among different demographic groups and help further elucidate specific risk factors.

Our study had several limitations. We used a convenience sampling, so not all regions were well represented, and sample size was relatively small. Our prevalence estimate may be inflated because positive predictive value is reduced in populations in which prevalence is low. The assay we used is the reference standard for clinical diagnosis but has not been used to test asymptomatic persons. Although an association between human *B. procyonis* exposure and seroconversion has not been established, asymptomatic seropositive infections would be expected because clinical disease probably occurs only when larvae cause damage to neural tissue or eyes (*1*). An estimated 95% of migrating larvae enter muscle or visceral organs, where they may stimulate an immune response but not cause clinical disease (*1*). In support of this presumption, the assay we used indicated that experimental infections of *Peromyscus* rodents with low numbers of *B. procyonis* parasites resulted in no clinical disease with seroconversion (S.G.H. Sapp, unpub. data). Last, participants were primarily licensed rehabilitators who belonged to professional organizations, and many practiced rehabilitation in large, dedicated facilities. Such facilities generally have safety protocols that may encourage more consistent PPE use and awareness of zoonotic diseases, so the risk for infection may be greater in smaller or informal rehabilitation settings.

To prevent infection with *B. procyonis* parasites, proper PPE and hand hygiene practices should be used consistently when handling animals and when contact with animal feces might occur. Education materials and outreach efforts discussing PPE use, infection control, and zoonotic pathogens should be directed to wildlife rehabilitators to increase awareness of potential occupational risks.

Technical Appendix Details regarding participant enrollment, acquisition of samples, serologic testing, and data analysis in a study of *Baylisascaris procyonis* roundworm seroprevalence among wildlife rehabilitators, United States and Canada, 2012–2015.

## References

[1] Kazacos KR. *Baylisascaris* larva migrans. Circular series no. 1412. Reston (VA): US Geological Survey; 2016.

[2] Cunningham CK, Kazacos KR, McMillan JA, Lucas JA, McAuley JB, Wozniak EJ, Diagnosis and management of *Baylisascaris procyonis* infection in an infant with nonfatal meningoencephalitis. Clin Infect Dis. 1994;18:868–72. 10.1093/clinids/18.6.868 8086545

[3] Cortez RT, Ramirez G, Collet L, Giuliari GP. Ocular parasitic diseases: a review on toxocariasis and diffuse unilateral subacute neuroretinitis. J Pediatr Ophthalmol Strabismus. 2011;48:204–12. 10.3928/01913913-20100719-02 20669882

[4] Hung T, Neafie RC, Mackenzie IR. *Baylisascaris procyonis* infection in elderly person, British Columbia, Canada. Emerg Infect Dis. 2012;18:341–2. 10.3201/eid1802.111046 22305101 PMC3310454

[5] Conraths FJ, Bauer C, Cseke J, Laube H. Arbeitsplatzbedingte Infektionen des Menschen mit dem Waschbärspulwurm (*Baylisascaris procyonis*). Arbeitsmed Sozialmed Umweltmed. 1996;31:13–7.

[6] Won KY, Kruszon-Moran D, Schantz PM, Jones JL. National seroprevalence and risk factors for Zoonotic *Toxocara* spp. infection. Am J Trop Med Hyg. 2008;79:552–7. 18840743

[7] Rascoe LN, Santamaria C, Handali S, Dangoudoubiyam S, Kazacos KR, Wilkins PP, Interlaboratory optimization and evaluation of a serological assay for diagnosis of human baylisascariasis. Clin Vaccine Immunol. 2013;20:1758–63. 10.1128/CVI.00387-13 24049107 PMC3837780

[8] Blizzard EL, Yabsley MJ, Beck MF, Harsch S. Geographic expansion of *Baylisascaris procyonis* roundworms, Florida, USA. Emerg Infect Dis. 2010;16:1803–4. 10.3201/eid1611.100549 21029553 PMC3294519

[9] Chavez DJ, LeVan IK, Miller MW, Ballweber LR. *Baylisascaris procyonis* in raccoons (*Procyon lotor*) from eastern Colorado, an area of undefined prevalence. Vet Parasitol. 2012;185:330–4. 10.1016/j.vetpar.2011.11.002 22119387

[10] Cottrell WO, Heagy RL, Johnson JB, Marcantuno R, Nolan TJ. Geographic and temporal prevalence of *Baylisascaris procyonis* in raccoons (*Procyon lotor*) in Pennsylvania, USA. J Wildl Dis. 2014;50:923–7. 10.7589/2014-02-032 25105813

[11] Pipas MJ, Page LK, Kazacos KR. Surveillance for *Baylisascaris procyonis* in raccoons (*Procyon lotor*) from Wyoming, USA. J Wildl Dis. 2014;50:777–83. 10.7589/2013-10-263 25014908

[12] Reiczigel J, Földi J, Ozsvári L. Exact confidence limits for prevalence of a disease with an imperfect diagnostic test. Epidemiol Infect. 2010;138:1674–8. 10.1017/S0950268810000385 20196903

[13] Amaral HLDC, Rassier GL, Pepe MS, Gallina T, Villela MM, Nobre MO, Presence of *Toxocara canis* eggs on the hair of dogs: a risk factor for Visceral Larva Migrans. Vet Parasitol. 2010;174:115–8. 10.1016/j.vetpar.2010.07.016 20728996

[14] Shafir SC, Sorvillo FJ, Sorvillo T, Eberhard ML. Viability of *Baylisascaris procyonis* eggs. Emerg Infect Dis. 2011;17:1293–5. 10.3201/eid1707.101774 21762591 PMC3381372

[15] Bauer C, Gey A. Efficacy of six anthelmintics against luminal stages of *Baylisascaris procyonis* in naturally infected raccoons (*Procyon lotor*). Vet Parasitol. 1995;60:155–9. 10.1016/0304-4017(94)00774-7 8644451

