# Supplementary material for: Baylisascaris procyonis Roundworm Seroprevalence among Wildlife Rehabilitators, United States and Canada, 2012–2015
Source: Emerg Infect Dis. 2016 Dec;22(12):2128–31. doi: 10.3201/eid2212.160467 (PMC5189140; doi:10.3201/eid2212.160467)
Supplement: Technical Appendix — Details regarding participant enrollment, acquisition of samples, serologic testing, and data analysis in a study of Baylisascaris procyonis roundworm seroprevalence among wildlife rehabilitators, United States and Canada, 2012–2015. [file 16-0467-Techapp-s1.pdf]

Article DOI: http://dx.doi.org/10.3201/eid2212.160467

Baylisascaris procyonis Seroprevalence among Wildlife Rehabilitators, United States and Canada, 2012–2015

Technical Appendix

Materials and Methods

Subject Enrollment and Sample Acquisition

During 2012–2015, attendees at regional or national wildlife rehabilitation professional meetings were asked to participate in the study (n = 303). The participants recruited at the professional meetings represented ≈30%–85% of the total attendance at these events; however, some individuals attended multiple meetings. A limited number of individuals (n = 44) who wanted to enroll in the study but were unable to attend the meetings obtained the consent form, questionnaire, and a sampling kit from the study staff and later provided serum for testing. Healthy, non-pregnant adults (at least 18 years of age) reporting contact with any wildlife species were eligible for inclusion. A 31-item questionnaire was administered assessing wildlife rehabilitation history and experience for all participants, and demographic information. Participants reporting raccoon contact in the past twelve months provided responses regarding the nature of their raccoon contact, husbandry practices, and personal protective equipment (PPE); including gloves, hand hygiene, and masks) use frequency in different scenarios. This questionnaire is available upon request.

Approximately 20 mL of blood was collected into blood collection tubes (Becton Dickinson, Franklin Lakes, NJ, USA) from each participant by a trained phlebotomist. Samples were allowed to clot and then centrifuged at 1,500 x g for 15 minutes. Serum was collected and stored at −20 C until testing. Serum samples and questionnaires were coded with a numerical identifier and the identification key was only available to one of the researchers (MJY). The recruitment and enrollment procedures and sample collection methods were reviewed and approved by the University of Georgia Institutional Review Board (MOD00002218).

Serologic Testing and Data Analysis

Sera were tested as previously described (*1*), with the following modifications: a control sample of pooled anti-*Toxocara* sera from confirmed visceral toxocariasis cases was run concurrently with each batch of samples tested to ensure that cross-reaction with *Toxocara* was not occurring, and commercially-produced (GenScript, Piscataway, New Jersey), *E. coli*-expressed, GST-tagged rBpRAG1 (*Baylisascaris procyonis* repeat antigen 1) was used for sample testing (n = 68) after December 2014 instead of in-house–produced antigen. A positive reaction was defined as a single band present at 37 KDa (63 KDa for GST-tagged antigen). Positive or ambiguous samples were tested in triplicate and read independently by two individuals for confirmation. An adjusted seroprevalence estimate considering assay performance characteristics and the associated confidence interval were calculated using R statistical software version 3.2.1 (*2*,*3*).

References

1. Rascoe LN, Santamaria C, Handali S, Dangoudoubiyam S, Kazacos KR, Wilkins PP, et al. Interlaboratory optimization and evaluation of a serological assay for diagnosis of human baylisascariasis. Clin Vaccine Immunol. 2013;20:1758–63. PubMed http://dx.doi.org/10.1128/CVI.00387-13

2. R Core Team. R: A language and environment for statistical computing. Vienna, Austria: R Foundation for Statistical Computing; 2014.

3. Reiczigel J, Földi J, Ozsvári L. Exact confidence limits for prevalence of a disease with an imperfect diagnostic test. Epidemiol Infect. 2010;138:1674–8. PubMed http://dx.doi.org/10.1017/S0950268810000385
